# Gene Therapy with HSV1-sr39TK/GCV Exhibits a Stronger Therapeutic Efficacy Than HSV1-TK/GCV in Rat C6 Glioma Cells

**DOI:** 10.1155/2013/951343

**Published:** 2013-03-03

**Authors:** Lei-qing Li, Fang Shen, Xiao-yan Xu, Hong Zhang, Xiao-feng Yang, Wei-guo Liu

**Affiliations:** ^1^Department of Intensive Care Unit, the Second Affiliated Hospital of Zhejiang University School of Medicine, Hangzhou 310009, China; ^2^Department of Neurosurgery, Huashan Hospital, Fudan University, Shanghai 200040, China; ^3^Department of Neurology, the Sir Run Run Shaw Hospital, Zhejiang University School of Medicine, Hangzhou 310016, China; ^4^Department of Nuclear Medicine, the Second Affiliated Hospital of Zhejiang University School of Medicine, Hangzhou 310009, China; ^5^Department of Neurosurgery, the First Affiliated Hospital of Zhejiang University School of Medicine, Hangzhou 310009, China; ^6^Department of Neurosurgery, the Second Affiliated Hospital of Zhejiang University School of Medicine, Hangzhou 310009, China

## Abstract

Although the combination of herpes simplex virus type 1 (HSV-1) thymidine kinase (TK) with ganciclovir (GCV) has been shown as a promising suicide gene treatment strategy for glioma, the almost immunodepressive dose of GCV required for its adequate *in vivo* efficacy has hampered its further clinical application. Therefore, In order to reduce the GCV dose required, we aim to compare the therapeutic efficacy of HSV1-sr39TK, an HSV1-TK mutant with increased GCV prodrug catalytic activity, with wildtype TK in C6 glioma cells. Accordingly, rat C6 glioma cells were first transfected with pCDNA-TK and pCDNA-sr39TK, respectively, and the gene transfection efficacy was verified by immunocytochemistry and western blot analysis. Then the *in vivo* sensitivity of these transfected C6-TK and C6-sr39TK cells to GCV was determined by 3-(4,5)-dimethylthiahiazo-(-z-y1)-3,5-di-phenytetrazoliumromide (MTT) colorimetric assay and Hoechst-propidium iodide (PI) staining. Finally, a subcutaneously C6 xenograft tumor model was established in the nude mice to test the *in vitro* efficacy of TK/GCV gene therapy. Our results showed that, as compared with wildtype TK, HSV1-sr39TK/GCV demonstrated a stronger therapeutic efficacy against C6 glioma both *in vitro* and *in vivo*, which, by reducing the required GCV dose, might warrant its future use in the treatment of glioma under clinical setting.

## 1. Introduction

Despite recent advances in neurosurgical techniques that facilitate more aggressive resection of tumor bulks and improvements in adjuvant radiochemotherapy, the prognosis of patients with high grade glioma is still extremely poor, with a median survival time of <1 year [1, 2], and only 8%–12% of patients survived more than two years [3]. However, the dramatic progresses in gene therapy in the past decades have drawn increasing interests in the application of suicide gene system as a new treatment strategy for glioma. 

Herpes simplex virus type 1 (HSV-1) thymidine kinase (TK) in combination with ganciclovir (GCV) has been shown as one of the most promising suicide gene systems for tumor treatment in laboratory studies and is currently being tested in several clinical trials. However, the GCV dose required for tumor ablation is almost immunosuppressive under clinical settings. An alternative strategy to reduce the GCV dose required is by increasing the catalytic activity of HSV1-TK on GCV, such as through mutagenesis of the active site of this enzyme [4–6]. HSV1-sr39TK is one of such promising mutants and has been reported to be endowed with a 14-fold decrease in Michaelis constants (*K*_*m*_) for GCV when compared with wildtype TK [5]. Enhanced tumor killing effect has also been demonstrated in tumors expressing HSV1-sr39TK, as compared with those expressing wildtype TK [4, 7–9]. However, in these studies, the therapeutic efficacies were mainly determined by using calipers to measure tumor sizes, while lacking *in vivo* evaluation on the early treatment responses of tumors to TK/GCV.

Positron emission tomography (PET) is a noninvasive imaging technique that allows quantitative *in vivo* analysis of the rates of various physiological and biochemical processes. Recently, improvement in scanner resolution has allowed microPET to become a potential method to monitor metabolic patterns in small animal models. Several PET molecular imaging agents could be used for monitoring tumor responses to prodrug activation in gene therapy. One of the most widely used agents in clinical setting is 2-[^18^F]-fluoro-2-deoxy-D-glucose (FDG) [10, 11], which is used as a marker of metabolic activity for glucose. FDG has been proved useful as a PET agent in oncology studies [12, 13] and has been widely used for monitoring the efficacies of anticancer strategies under clinical setting. Numerous studies have demonstrated that changes in FDG level in response to treatment correlates with subsequent clinical and radiological response [11]. In the present study, we used FDG microPET as an *in vivo* evaluation method to assess the early therapeutic efficacy of the suicide gene therapy. Consequently, there were two objectives in this study. One was to determine whether the mutant TK, as compared with wildtype one, could exhibit a stronger glioma inhibition effect. The other was to determine the value of microPET imaging in the assessment of the tumor responses to suicide gene therapy.

## 2. Materials and Methods

### 2.1. Cell Lines

The rat C6 cell line was supplied by Shanghai Institute of Biochemistry and Cell Biology and was cultured as monolayers in DMEM (GIBCO, Grand Island, New York, USA) supplemented with 10% fetal calf serum and 1% penicillin streptomycin. Cells were cultured in a humidified atmosphere with 5% CO_2_ at 37°C and were routinely passaged by trypsinization with a change of medium twice weekly. 

### 2.2. Vector Construction

Plasmid PNGVL expressing HSV1-TK (PNGVL-TK, [14]) was a kind gift from Dr. Joseph Ciccolini (School of Pharmacy, Marseille, France). The HSV1-TK gene was cloned from PNGVL-TK into empty pCDNA3.1 (Invitrogen/Gibco, Grand Island, New York, USA) by the polymerase chain reaction (PCR), using the forward primer 5'-TGT GAA TTC CCA CCA TGG CTT CGTA-3' and the reverse primer 5'-GAC GCT CGA GTA AGT CAG TTA GCC TCC-3'. The initial denaturation at 94°C for 4 min was followed by 35 cycles at 94°C for 50 sec, 60°C for 50 sec, and 72°C for 1 min. The PCR product was cleaved with Xho I and Eco R I, gel purified, and ligated into the Xho I and Eco R I sites of pCDNA3.1 to yield pCDNA-TK. The accuracy of the HSV1-TK gene sequence in pCDNA-TK and HSV1-sr39TK gene sequence in plasmid pCDNA-sr39TK (kindly supplied by Pro. Gambhir, Stanford University, CA, USA) were further confirmed by DNA sequencing. 

### 2.3. Cells Transfection

Effectene transfection reagent kit (buffer EC, Enhancer and Effectene reagent) was purchased from Qiagen company (Qiagen China Co., Ltd., Shanghai, China). Before transfection, 2 × 10^5^ C6 cells were seeded into six-well plate and allowed to proliferate until 70%–80% of the cells were confluent. Shortly before transfection, the culture medium was replaced with 1 mL fresh complete medium. Plasmid DNA (0.4 *μ*g) was first mixed with the DNA-condensation buffer (buffer EC) to a final volume of 60 *μ*L, and then incubated with 3.2 *μ*L Enhancer at room temperature for 5 min. After incubation with 10 *μ*L Effectene reagent for another 10 min, the Effectene-DNA complex was mixed with 1 mL fresh medium, and then added to the cells, which was then incubated at their normal growth condition as mentioned above for another 48 hours. 

### 2.4. Immunocytochemistry for HSV1-TK Expression

Expression of HSV1-TK or HSV1-sr39TK gene was tested immunocytochemically with the HSV1-TK-antibody. After antigen repaired with EDTA buffer at 80°C and washed with phosphate buffered saline (PBS) for three times, cell slices were incubated in 10% rabbit serum for 10 min for antigen blocking and then incubated with goat polyclonal anti-HSV1 TK (1 : 200 dilution; Santa Cruz Biotechnology, Inc., Santa Cruz, CA, USA) for 2 hours at 37°C. After being washed with PBS for three times, the slices were incubated with rabbit anti-goat biotinylated IgG (Beijing Zhongshan Golden Bridge Biological Technology Co., Ltd., Beijing, China) and streptavidin (Beijing Zhong Shan-Golden Bridge Biological Technology Co., Ltd., Beijing, China) for 15 min, respectively, at 37°C. Diaminobenzidine was used as the chromogen, which showed a brown stain in areas with HSV1-TK or HSV1-sr39TK expression. Slices were then counterstained with hematoxylin. After differentiation, dewatering with gradient alcohol and mounted with neutro-resina, the slices were then observed under optical microscope.

### 2.5. Western Blot Analysis

Cell protein was separated by electrophoresis. After blocking with TBS-Tween (TBS with 0.05% Tween) containing 5% nonfat dry milk, the membranes were incubated with goat polyclonal anti-HSV1 thymidine kinase (1 : 200 dilution in TBS-Tween containing 5% nonfat dry milk) at 4°C over night. After being washed with TBS-Tween, the membranes were incubated for 2 hours at room temperature with an HRP-conjugated secondary antibody (1 : 5000 dilution in TBS-Tween containing 5% nonfat dry milk; Santa Cruz Biotechnology, Inc., Santa Cruz, CA, USA). Membranes were washed again with TBS-Tween, and protein bands were visualized by Luminol Reagent (Santa Cruz Biotechnology, Inc., Santa Cruz, CA, USA) according to the manufacturer's guidelines. Exposure times of membranes on Kodak Medical X-ray films ranged from 30 to 60 sec. After being washed in stripping buffer (2% SDS, 62.5 mmol/L Tris-HCl, 100 mmol/L *β*-mercaptoethanol) for 30 min at 50°C, the PVDF membrane was rinsed with TBS-Tween and exposed again to *β*-actin antibody (1 : 2000, Santa Cruz Biotechnology, Inc., Santa Cruz, CA, USA) as an internal standards control. The bands on the films were scanned and analyzed with Scion Image software (Scion Corporation, Frederick, MD, USA). The ratio between the target proteins and *β*-actin band densities was used for semiquantitative evaluation of the concentrations of the target proteins.

### 2.6. *In Vitro* GCV Sensitivity Assay

3-(4,5)-dimethylthiahiazo-(-z-y1)-3,5-di-phenytetrazoliumromide (MTT) colorimetric assay: the sensitivity of C6-TK and C6-sr39TK to GCV (Hubei Keyi Pharmaceutical Co., Ltd., Hubei, China) was determined *in vitro* using eight different GCV concentrations: 0 *μ*M, 4 *μ*M, 8 *μ*M, 20 *μ*M, 40 *μ*M, 80 *μ*M, 200 *μ*M, and 400 *μ*M. On day 1, control C6, C6-TK, and C6-sr39TK cells were planted in 96-well plates with a density of 5000 cells/well. Growth medium containing the corresponding GCV concentrations was added 24 hours later. After 72 hours of GCV treatment, cell survival rate (SR) was determined using routine MTT method, and absorbance (*A*) values were read at 570 nm wavelength. SR was calculated as follows: SR = ((*A*  value  of  test  well)/(*A*  value  of  the  control)) × 100%.

Hoechst-propidium iodide (PI) staining assay: cells were seeded into six-well plates and stained by Hoechst 33342 and PI 72 hours after 400 *μ*M GCV administration. The percentages of PI positive cells of each group were counted under a fluorescence microscope. Nucleus morphological analysis of Hoechst positive cells was also performed under the fluorescence microscope.

### 2.7. *In Vivo* Studies

One million untransduced C6 cells were injected subcutaneously into the left flank of 20 male nude mice (BALB/c, 5 to 6 weeks, 18–20 g, supplied from Experimental Animal Center of Zhejiang University School of Medicine, Hangzhou, China) as control, which were then randomly assigned to two groups (*N* = 10/group) and inoculated subcutaneously with one million C6-TK or C6-sr39TK cells in the right frank (day 0). One week later (day 7), prodrug GCV was administrated by intraperitoneal injection (100 mg/kg) twice a day for 10 consecutive days (day 7–day 16). Antitumor effect was evaluated by measuring the long (*a*) and the short (*b*) axes of the coronal plane of each tumor, which yielded the maximal area, by a caliper at day 7 (before treatment) and day 17 (after treatment). The tumor volume (V) was calculated according to the following formula: V  (mm^3^) = *a* × *b* ^2^ × *π*/2 [15]. Tumor growth inhibition rate by day 17 was calculated as follows: (1 − V_transfection group_/V_control group_) × 100%.

All the animals were sacrificed at day 17, and tumor tissues were dissected and fixed in 4% formaldehyde for 48 hours and then embedded in paraffin. Four *μ*m sections were prepared and stained with hematoxylin and eosin (HE). All experiments were approved by local ethnic committee and carried out according to the Guidelines to the Care and Use of Laboratory Animals of Zhejiang University (Ethics Code: no. ZJU2006-1-02-032).

### 2.8. MicroPET Studies

At day 7 and day 17 during the *in vivo* studies (corresponding to before and after GCV administration), all the animals received microPET scans. PET was performed using a microPET R4 rodent model scanner (CTI Concorde Microsystems, Knoxville, TN, USA), which was equipped with a microPET manager for data acquisition in the list mode and Acquisition Sinogram and Image Processing (ASIPro) for preparing sinograms and image reconstruction. The scanner had a computer-controlled bed, a 10.8 cm transaxial, and a 7.8 cm axial field of view (FOV) with intrinsic image resolution of <1.8 mm. FDG was prepared with a specific activity of 500 Ci/mmol at the Department of Nuclear Medicine, Zhejiang University School of Medicine, using an automated FDG synthesis system (Hamamatsu Photonics CO, Nishi Ward, Hamamatsu, Japan) according to the instructions.

Before the scans, all the mice were anesthetized by i.p. injection of 200 *μ*L of pentobarbital sodium solution (5 mg/mL, i.e., 50 mg/kg) and then were injected with FDG (7.4 MBq, 200 *μ*Ci) through the tail vein. Half an hour later [16], the mice were placed at the center of the FOV of the microPET R4 scanner at a spread prone position and underwent a 10 min static scan. Images were reconstructed by a maximum-a-posteriori probability (MAPP) algorithm. Regions of interests (ROI) were manually drawn around the edge of the tumor xenograft from the sequential coronal slices of PET images where the tumor was visible. Besides, ROIs were also drawn around the left shoulder with the same size as the corresponding tumor on the the same image slices. Ratio of radioactivity in the tumor tissue to the muscle of each slices was calculated using ASIPro. 

### 2.9. Statistical Analysis

Results were expressed as means ± standard deviation (SD). Statistical analysis was conducted by the one-way analysis of variance (ANOVA) and *χ* ^2^ test for comparisons among multiple groups or between two groups using the SPSS 13.0 software package, and a *P* value less than 0.05 was considered statistically significant. When analysis of variance identified a significant difference (*P* < 0.05) between the groups, each group was compared with the Student-Newman-Keuls (SNK) test to identify which 2 groups were statistically different (*P* < 0.05).

## 3. Results

### 3.1. Expression of Mutant and Wildtype TK Gene in C6 Cells

**Figure 1:**
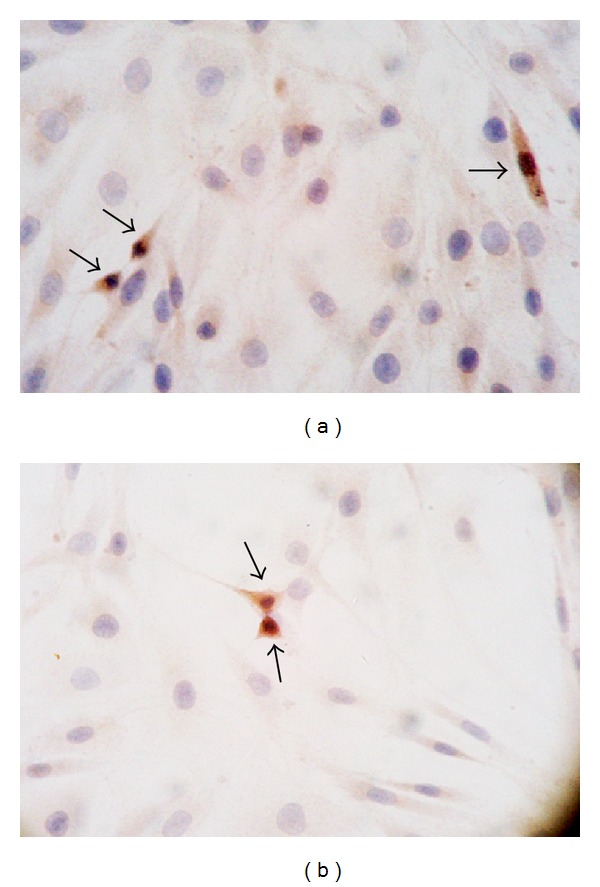
Determination of HSV1-TK and HSV1-sr39TK gene expression level in C6 cells by immunocytochemistry 48 hours after addition of DNA-liposome complex (×400). The percentages of TK positive cells in cultured C6-TK (a) and C6-sr39TK cells (b) were 13.25 ± 1.12% and 13.76 ± 2.09%, respectively (*P* > 0.05). Black arrow indicated immunostaining positive cells.

Forty-eight hours after the addition of DNA-liposome complex, immunocytochemistry analysis demonstrated a heterogeneous HSV1-TK expression in both C6-TK and C6-sr39TK cells (Figure 1), whereas no positive cell was found in control C6 cells. The transduction efficiency in cultured C6-TK and C6-sr39TK was 13.25 ± 1.12% and 13.76 ± 2.09%, respectively, with no significant difference between the two groups (*χ* ^2^ = 0.142, *P* = 0.706).

The expression of recombination proteins in target cells was further confirmed by western blot, in which a distinctive band of about 44 KD was observed on PVDF membrane. This band corresponds to the predicted molecular weight of HSV1-TK and HSV1-sr39TK, while the control C6 cells had no similar bands. Semiquantitation analysis revealed that there was no significant difference (*P* = 0.095) between C6-TK (0.26 ± 0.01) and C6-sr39TK (0.23 ± 0.02) group in their TK gene protein expression level.

### 3.2. *In Vitro* Sensitivity of Transfected C6 Cells to GCV

**Figure 2:**
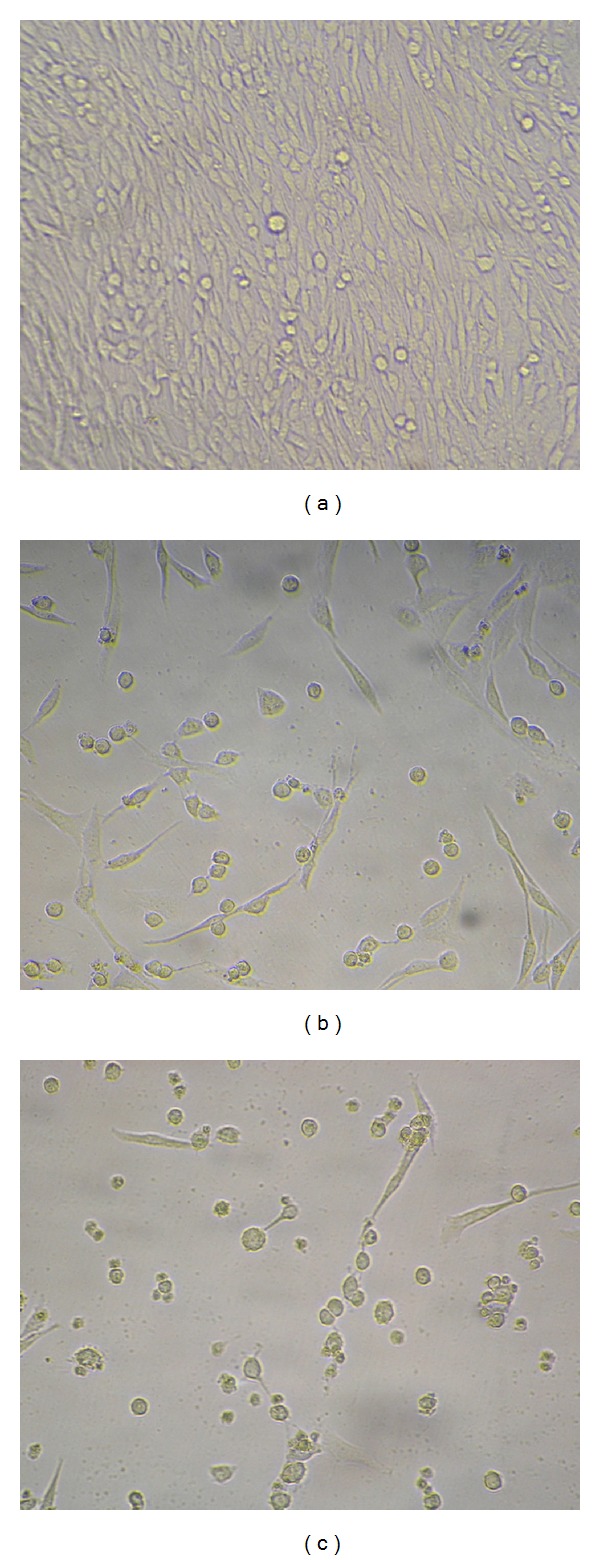
Morphological changes of C6-TK and C6-sr39TK cells 72 hours after ganciclovir (GCV) administration (×200). As compared with control C6 cells (a), C6-TK (b) and C6-sr39TK cells (c), especially the latter, showed evident morphological changes that were characteristic of growth inhibition/cellular death after GCV treatment: cells became round and shrunk, lost their protuberances, and detached from the floor of culture flasks.

**Figure 3:**
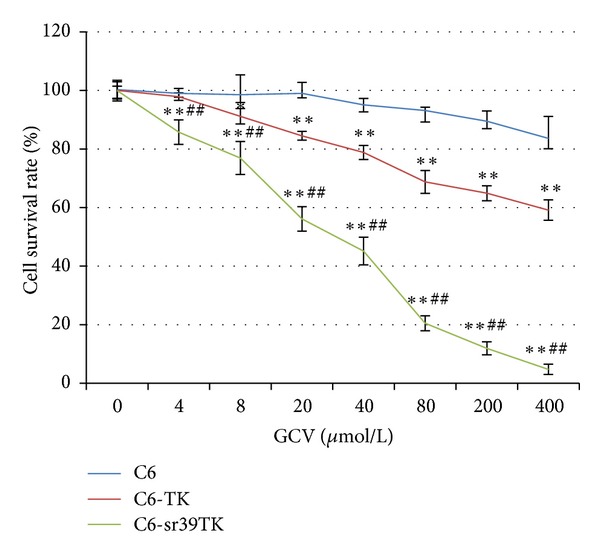
Growth inhibition in cultured C6-TK and C6-sr39TK cells 72 hours after ganciclovir (GCV) administration. Cell survival rate (SR) was measured by MTT assay, which showed SR in C6-TK and, especially, the C6-sr39TK group was significantly decreased in a dose-dependent manner as compared with that of the control (*P < 0.05  versus  C6 group; **P < 0.01  versus  C6 group; ^##^ *P* < 0.01  versus  C6-TK group).

With increase in GCV concentration, C6-TK and C6-sr39TK, especially the latter, presented evident morphological changes and were characterized with growth inhibition and cell death: cells became round and shrunk, lost their protuberances, detached from the floor of culture flasks, and finally died (Figure 2). The SR after 72 hours of GCV administration in each group was shown in Figure 3, which showed that SR in C6-TK and C6-sr39TK group was significantly decreased in a dose-dependent manner as compared with that of the control (*P* < 0.05). And the C6-sr39TK cells were most sensitive to GCV, with a half inhibitory concentration (IC_50_) of 25.35 *μ*M. In contrast, the SR of control C6 was not significantly affected by the presence of GCV when the concentration was less than 400 *μ*M. The IC_50_ of GCV for C6-TK was 399.20 *μ*M, which was about 15-fold higher than that of C6-sr39TK.

**Figure 4:**
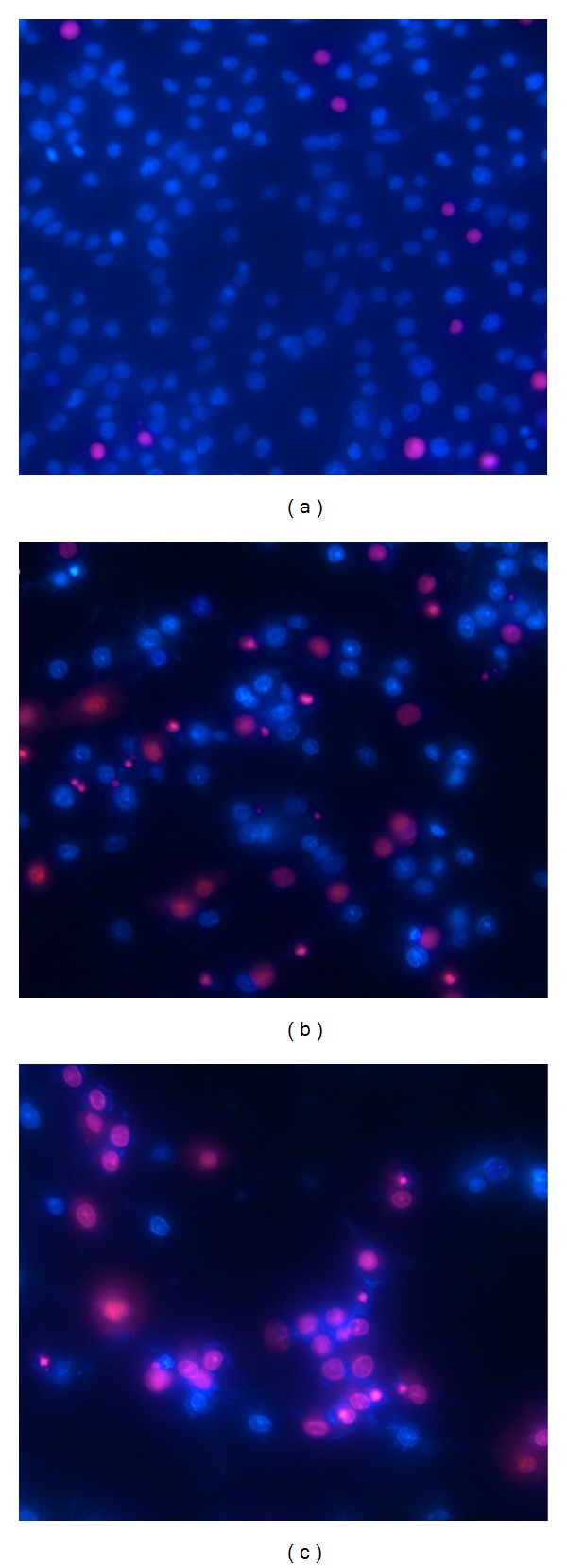
Propidium iodide (PI) and Hoechst 33342 double staining in cultured C6 cells 72 hours after 400 *μ*M ganciclovir (GCV) administration (×400). Cells emitting blue fluorescence were Hoechst positive while those with red fluorescence were PI positive. Although PI positive cells (dead cells) were rare in control group (a), their number increased significantly in C6-TK (b) and C6-sr39TK group (c) (C6-TK versus C6-sr39TK, *P* < 0.001).

Double labeling with Hoechst 33342 and PI 72 hours after 400 *μ*M GCV administration showed that most cells in control group were Hoechst positive, as their nuclei uniformly and hazily emitted blue fluorescence, while the dead cells, which emitted red fluorescence instead, were rarely found. The percentage of PI positive cells increased significantly in both C6-TK and C6-sr39TK group (31.53 ± 0.02% and 60.96 ± 0.02%, resp.), and the difference was significant (*χ* ^2^ = 35.765, *P* < 0.001). Among the Hoechst positive cells, some features characteristic of apoptosis, for example, condensed or fragmented nuclei, were observed. Representative Hoechst-PI staining was shown in Figure 4.

### 3.3. *In Vivo* Inhibition of Xenograft Tumors by GCV

In the *in vivo* models, all mice developed xenograft tumors 5 to 7 days after cell inoculation, and the tumor volume ranged from 266.32 to 449.91 mm^3^. The average tumor sizes in control C6, C6-TK, and C6-sr39TK groups before GCV treatment were 348.68 ± 61.55, 345.74 ± 54.72, and 374.07 ± 70.94 mm^3^, respectively, with no significant difference among them (*F* = 0.174, *P* > 0.05).

**Figure 5:**
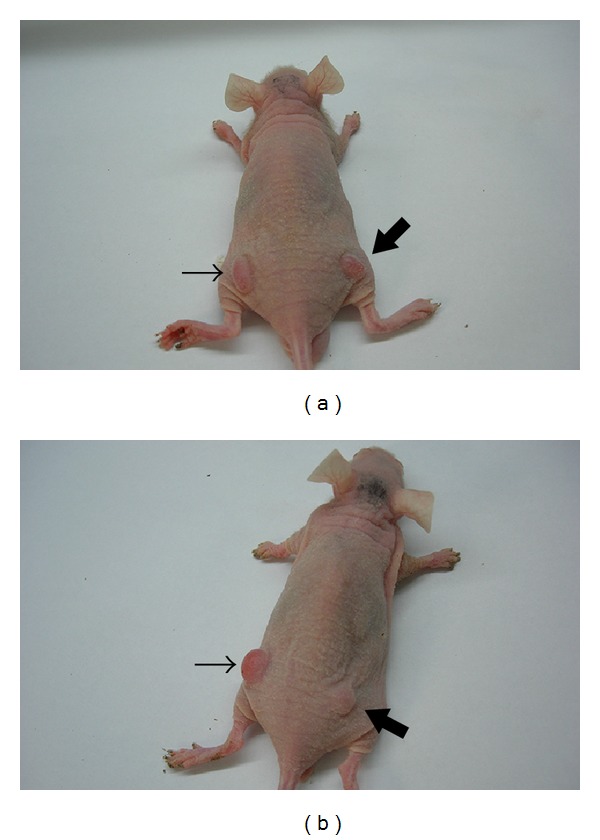
Representative images showing ablation of subcutaneous xenograft tumors by intraperitoneal ganciclovir (GCV) administration in nude mice. Thin arrow indicated xenograft tumors derived from control C6 cells, while thick arrow represented tumors that originated from either C6-TK cells (a) or C6-sr39TK cells (b). Although complete tumor ablation was not achieved, tumors in the mutant TK groups were significantly smaller than that from the wildtype TK or control group.

After a 10-day treatment with GCV following tumor formation, the average volume of xenograft tumors developed from C6-sr39TK, C6-TK, or C6 cells were 574.08 ± 107.72, 928.47 ± 165.61, and 1287.24 ± 364.84 mm^3^, respectively, with a significant difference among these groups (*P* < 0.05) (Figure 5). And post hoc SNK multiple comparisons demonstrated that statistically significant differences existed between C6-sr39TK and C6-TK group (*q* = 4.017, *P* < 0.01). Although neither wildtype TK nor mutant TK could completely eradicate the xenografts, the HSV1-sr39TK group showed a 38.17% or 55.40% reduction in tumor growth after GCV treatment as compared with wildtype TK or the control group, respectively. 

### 3.4. FDG Metabolic Profile of Xenograft Tumors

**Figure 6:**
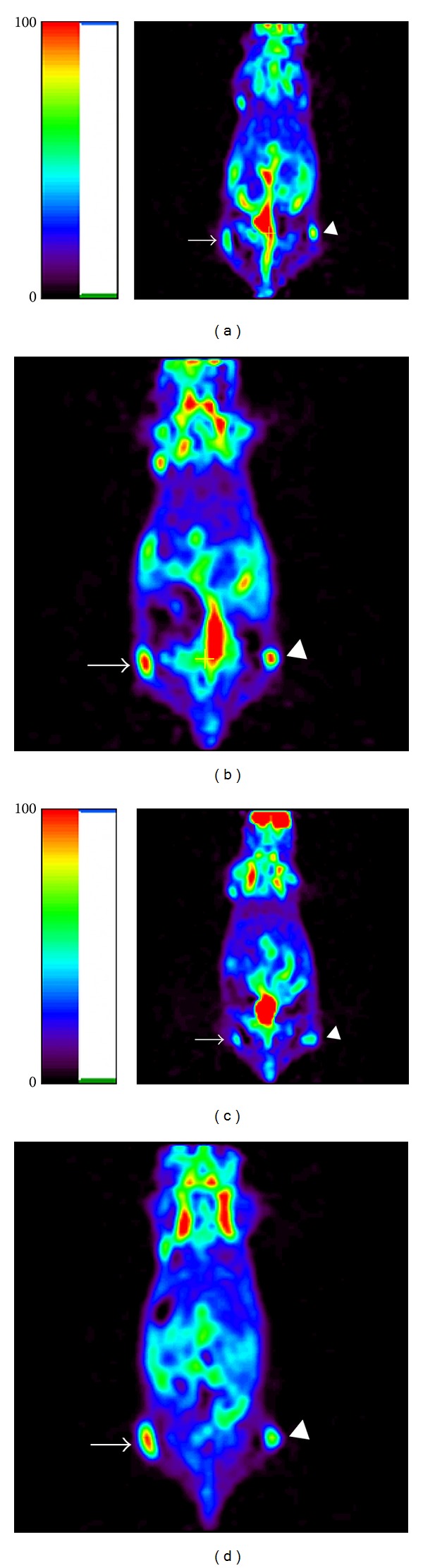
Representative microPET images before ganciclovir (GCV) administration ((a) and (c)) and by the end of experiment (10 days after GCV administration) ((b) and (d)). Arrow represented control C6 xenograft tumor and triangle represented C6-TK xenograft tumor in (a) and (b) and C6-sr39TK xenograft tumor in (c) and (d), respectively. SNK analysis revealed that the ratio of radioactivity (tumor/muscle) in C6-sr39TK and C6-TK group was significantly lower than the control group (*P* < 0.05), but not between them (*P* > 0.05).

MicroPET scan showed that there was no significant difference in the average ratio of radioactivity (tumor/muscle) among control C6, C6-TK, and C6-sr39TK groups (1.71 ± 0.40 versus 1.82 ± 0.40 versus 1.62 ± 0.48, *F* = 0.150, *P* = 0.861) before GCV treatment. However, after 10-day GCV administration, a significant difference was found among these groups (*F* = 7.006, *P* = 0.003). To determine where exactly these differences existed, we further examined the results with SNK multiple comparisons. SNK analysis revealed that the ratio of radioactivity (tumor/muscle) in C6-sr39TK (3.34 ± 0.70) and C6-TK (3.65 ± 0.74) groups were significantly lower than the control group (4.58 ± 1.10) (*q* = 4.788, *P* < 0.05, and *q* = 3.601, *P* < 0.05, resp.), but not between C6-TK and C6-sr39TK groups (*q* = 1.054, *P* > 0.05). Representative FDG-microPET images were shown in Figure 6.

Interestingly, in wildtype TK group and the mutant TK group, additional scans performed in 6 mice at 4 and 7 days after GCV treatment demonstrated that the FDG accumulation in tumors increased at the early phase of GCV administration and then demonstrated a trend to decrease. No similar pattern in FDG metabolism was found in the control, which, despite GCV treatment, showed an increasing accumulation of FDG during the whole period. 

### 3.5. Pathological Examination

**Figure 7:**
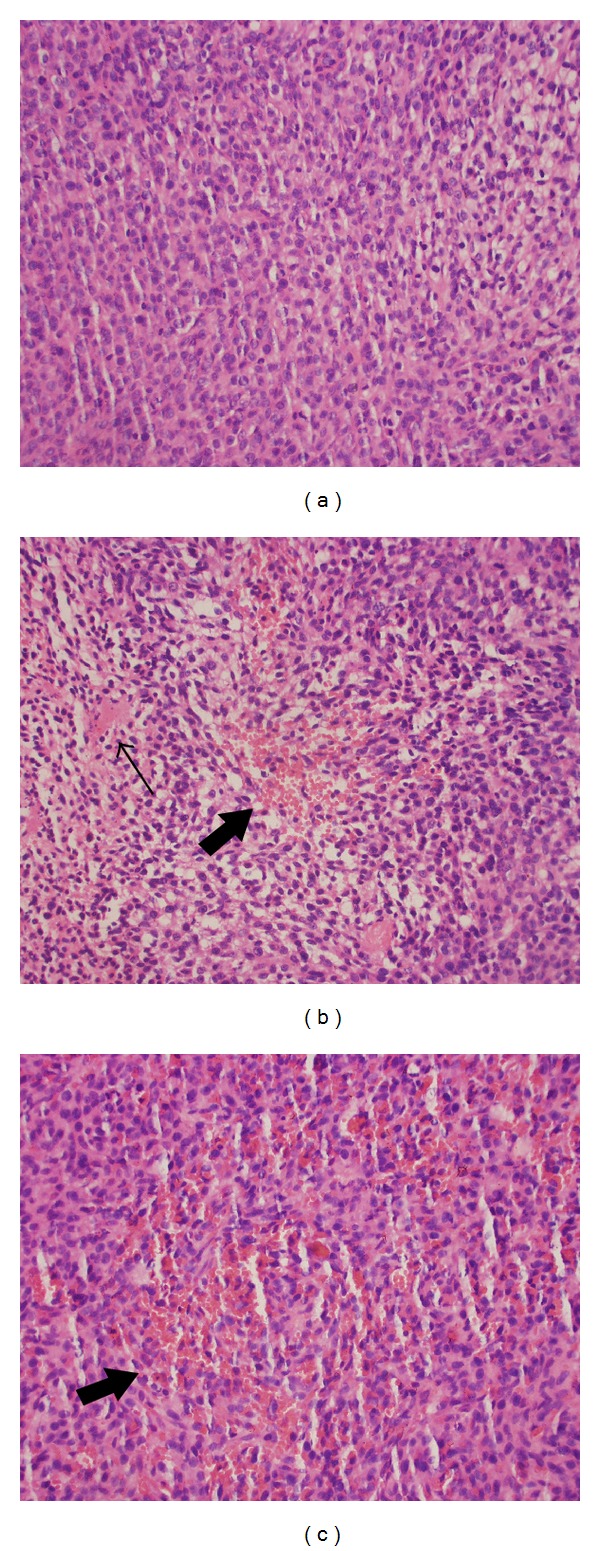
Hematoxylin and eosin (HE) staining of the xenograft tumor tissues from control group (a), C6-TK group (b), and C6-sr39TK group (c) at day 17 after ganciclovir (GCV) administration (×400). Thin arrow and broad arrow indicated necrotic or hemorrhagic areas in tumors from C6-TK group and, more prominently, the C6-sr39TK group.

HE staining verified that the xenograft tumors samples from all groups had morphological features characteristic of glioma. However, only tumors from the C6-TK and C6-sr39TK groups, especially the latter, exhibited a prominent necrotic and hemorrhagic changes, while samples from the control group did not. Representative pictures of HE staining were shown in Figure 7.

## 4. Discussion

The development of an effective therapy for gliomas remains a major challenge for neurooncologists. Despite advances in neurosurgical techniques, radiation treatment, and adjuvant chemotherapy, patients with glioma (which constitutes 40%–50% of primary brain tumors) still expect a poor prognosis, especially in those with high grades ones (WHO grades III-IV). The reason for this gloomy prognosis is partly due to the fact that glioma cells could aggressively infiltrate the surrounding normal brain parenchyma, therefore, making total tumor removal almost impossible [17]. In order to eradicate this formidable neoplasm, several gene therapy strategies have been developed, including transfection of suicide genes, tumor-suppressor genes, drug-sensitizing genes, and genes that enhance immunogenicity [18]. The most widely used gene therapy approach is the transfection of suicide gene, such as the HSV1-TK/GCV system [19–23], in which GCV is first phosphorylated by HSV1-TK to its monophosphates (GCV-MP) and further phosphorylated by cellular kinase to its triphosphates (GCV-TP). GCV-TP is highly cytotoxic because it can inhibit DNA polymerases and incorporate into DNA double strands [24–27], which cause DNA damages and finally lead to cell death. There are reports that show that the incorporation of GCV-MP into the host genome may also have something to do with the cytotoxicity caused by HSV1-TK/GCV [28].

To date, several clinical studies utilizing HSV-TK suicide gene system to treat cancer have been performed, but with varying results [19, 21, 22, 29–33]. The major reason for yielding negative treatment responses may be the inefficiency of the vector in transfecting the suicide gene to the host cancer cells and low HSV-TK expression level. Another major obstacle is that the GCV dose required to inhibit* in vivo* tumor progression was immunosuppressive [4]. In recent years, much efforts have been made to improve the efficacy of TK suicide gene therapy, that is, enhancing tumor cell killing effects without increasing prodrug-mediated toxicity in normal cells. One important approach is to construct novel HSV-TK gene mutants with increased prodrug phosphorylating capacity. From a random sequence derived library with over 1 million TK genes variants [34] and a semirandom library based on the amino acid modifications of the candidate TK mutants screened from the first library [4], several potential mutants have been reported with improved prodrug processing activities. With GCV as the substrate, further kinetic analysis [5] suggested one such mutant (HSV1-sr39TK), containing five amino acid modifications (L159I+I160F+F161L+A168F+L169M), as the mutant with best kinetic performance of GCV (14-fold decrease in *K*_*m*_ value as compared with the wildtype enzyme), and therefore turned out to be a more effective and much safer alternative to other TK mutants and wildtype ones. In this present study, our results have distinctively confirmed that transfection of HSV1-sr39TK could produce an enhanced tumor killing capacity of GCV against rat glioma C6 cells both *in vitro* and* in vivo*. 

In the present study, we have deployed several approaches to determine the treatment response of C6 glioma cells to suicide gene therapy. *In vitro*, two different cytotoxicity assays, namely, MTT and Hoechst-PI staining, were used to measure the cell viability after GCV treatment. The results of the two assays complemented each other and indicated an increased cytotoxicity of GCV in cells transfected with HSV1-sr39TK, as compared with that expressing wildtype TK; however, the extent of the *in vitro* differences between the TK mutant and the wildtype one (i.e., about 15-fold decreases in IC_50_) was different from those reported previously. For example, previous studies reported an approximately 300-fold lower IC_50_ in C6 cells transduced with HSV1-sr39TK versus wildtype TK [4], or about 100-fold decrease in IC_50_ [9] or 5-fold decrease in IC_50_ [8]. Different vectors used and different gene transduction efficiencies may be the two reasons responsible for the variances in these studies. We next set out to compare the *in vivo* treatment efficacies of the mutant gene therapy system with the wildtype one in nude mouse xenograft model. Although complete tumor eradication was not achieved, a much more significant tumor growth inhibition was found in HSV1-sr39TK expressing tumors. Besides, pathological examination of the xenograft tumor samples also confirmed a more pronounced tumor killing capacity in the mutant TK group, as indicated by more foci of hemorrhagic/necrotic changes.

However, as it is known for solid tumors, a reduction in tumor size by a specific therapy may take considerable time, and treatment responses at histopathological level usually can only be measured by the end point of treatment after sampling the tumor tissues [10]. In contrast, *in vivo* noninvasive evaluation of FDG metabolism by microPET was able to monitor and compare the tumor treatment responses at any desired time point during the whole treatment course. To the best of our knowledge, it was the first time to compare the therapeutic efficacy of mutant TK with wildtype TK in terms of processing GCV prodrug in C6 glioma xenografts by metabolism imaging using microPET. FDG microPET scans were performed before GCV administration and 10 days after drug administration. In the baseline scans, there was no significant differences in terms of FDG uptake among the groups. Ten days after GCV administration, there was a marked decrease in the metabolic activity in C6-TK and C6-sr39TK tumors, as compared with the control. Although the most dramatic change in tumor FDG uptake was observed in mutant TK, the difference between the mutant TK and the wildtype one was of no statistical significance. Although we could not therefore abruptly conclude that FDG microPET was not suitable for *in vivo* evaluation of the efficacy of suicide gene therapy, the molecular pathway mediating the changes in tumor FDG uptake after gene therapy may still need further studies. Scans performed at 4 and 7 days after GCV administration demonstrated the FDG uptake in C6-TK and C6-sr39TK group increased at the early posttreatment phase, decreased later, and remained above the baseline level by 10 days after treatment. Therefore, a scan interval of more than 10 days may be more suitable to measure the treatment efficacy. Increased FDG uptake in tumor cells early after therapeutic intervention was found both *in vitro* and *in vivo *[35, 36]. It has been suggested that this change was mainly due to enhanced glucose transport across the cell membrane [37], which may be caused by redistribution of the glucose transporter as a result of cellular stress after treatment [38]. 

There were also several limitations in this work. First, we used *in vitro* transfected C6 cells for *in vivo* study. However, microinjection of vectors containing target gene fragments through vein or directly into tumor bulks seems to be more suitable in real clinical settings. Second, the *in vivo* TK expression levels in the tumors were not examined, which might be solved by using reporter gene imaging system. For example, when used as a reporter gene for PET imaging, the mutant TK was confirmed to improve GCV cytotoxicity as compared with wildtype one [39]. 

To conclude, we have provided both *in vitro *and *in vivo* evidences supporting that an enhanced glioma inhibition effect could be achieved by introducing a novel HSV1-TK mutant, namely, HSV1-sr39TK. HSV1-sr39TK, by enhancing the cytotoxic effects and reducing the dose-related side effects of GCV, might be a promising candidate gene in future clinical suicide gene therapy for gliomas. Although FDG PET imaging failed to distinguish the difference in treatment responses between HSV1-TK and HSV1-sr39TK gene therapy in the present study, it remains an important noninvasive approach to monitor the early treatment responses of tumors to gene therapy in small animal models. However, the molecular mechanism underlying the changes in tumor FDG uptake after gene therapy and the practical value of FDG PET in the determination of treatment responses to suicide gene therapy still need further investigations.
